# At the end of the rope: *Geophilus hadesi* sp. n. – the world’s deepest cave-dwelling centipede (Chilopoda, Geophilomorpha, Geophilidae)

**DOI:** 10.3897/zookeys.510.9614

**Published:** 2015-06-30

**Authors:** Pavel Stoev, Nesrine Akkari, Ana Komerički, Gregory D. Edgecombe, Lucio Bonato

**Affiliations:** 1 Pensoft Publishers and National Museum of Natural History, Sofia, Bulgaria; 2 Naturrhistorisches Museum Wien, Burgring 7, 1010 Wien, Austria; 3 Croatian Biospeleological Society, Zagreb, Croatia; 4 Department of Earth Sciences, Natural History Museum, Cromwell Road, London SW7 5BD, UK; 5 Department of Biology, University of Padova, via U. Bassi 58b, I-35131 Padova, Italy

**Keywords:** *Geophilus*, troglomorphism, cave-dwelling species, extreme habitats, Velebit Mountain, Croatia

## Abstract

A new geophilomorph centipede, *Geophilus hadesi* sp. n., is described from caves in the Velebit Mountain, central Croatia. Together with *Geophilus persephones* Foddai & Minelli, 1999, described from Pierre Saint-Martin cave in France, they are the only two remarkably troglomorphic geophilomorphs hitherto known. The new species apparently belongs to a group of *Geophilus* species inhabiting mainly Western and Southern Europe, with a uniquely modified pretarsus in the second maxillae. *Geophilus hadesi* **sp. n.** shows unusual traits, some of which commonly found in troglobitic arthropods, including exceptionally elongated antennae, trunk segments and leg claws. The species is described upon specimens found in two caves at a depth below -250 m. Another two specimens apparently belonging to the same species have been recorded in another deep vertical cave at -980 m and -1100 m. The latter represents the world’s deepest record of Chilopoda as a whole.

## Introduction

Centipedes are common cave inhabitants. However, most species find shelter there only occasionally, thus representing trogloxenes or troglophiles at most. True troglobites – species with an entire life cycle confined to cave environments – are much rarer in the group (see review of cave myriapods by Culver and Shear (2012)). Such species are usually pallid, eyeless (or with reduced numbers of ocelli) and with long appendages that bear elongated setae, characters which have evolved in relation to the underground lifestyle. Other troglomorphic characters known for lithobiomorph centipedes include enlargement of Tömösváry’s organ and coxal pores.

Troglobites are currently described only in three out of the five extant orders of Chilopoda, namely Lithobiomorpha, Scolopendromorpha and Geophilomorpha, and an instance has been recorded in Scutigeromorpha as well (Howarth and Stone 1990; GDE, pers. obs.). However, they are not equally represented in these orders: lithobiomorphs seem much more prone to penetrate and adapt to this environment than the members of the other two orders, where only a handful of species are known (most of which belong to the blind scolopendromorph genus *Cryptops* Leach, 1815). Troglobitic centipedes are mainly known from the Northern Hemisphere, in particular southern Europe (the Balkan peninsula, the Pyrenees and Sardinia), in areas with vast coverage of limestone and high number of caves. Few troglobites are known from elsewhere, with only isolated examples south of the equator (Negrea and Minelli 1994; Edgecombe 2005, 2006; Ázara and Ferreira 2013, 2014a, b).

More than twenty years have elapsed since Negrea and Minelli (1994) provided a global overview of the cave-inhabiting centipedes. During that period, active biospeleological work has been carried out in several poorly explored regions of the world, delivering remarkable novelties. To name a few, the first troglomorphic geophilomorph was discovered in the Pyrenees (Foddai and Minelli 1999). Highly cave-adapted species of the genus *Cryptops* – the first descriptions of troglobitic centipedes on their respective continents – were found from caves in Australia (Edgecombe 2005, 2006) and Brazil (Ázara and Ferreira 2013, 2014a). New troglobitic species of Scolopocryptopidae (Scolopendromorpha) from the genera *Newportia* Gervais, 1847 and *Scolopocryptops* Newport, 1844 were described from Mexico (Chagas-Júnior and Shelley 2003), Puerto Rico (Schileyko 2013) and Brazil (Ázara and Ferreira 2014b, Chagas-Júnior and Bichuette 2015). New troglobitic species of Lithobiomorpha were found recently in Sardinia (Zapparoli 1997), Corsica (Iorio 2010), and North Africa (Stoev et al. 2010). Furthermore, the unique Movile cave in Romania was reported to harbor a troglobitic population of the common European centipede *Cryptops anomalans* (Negrea, 1993), a phenomenon which still needs to be further explored, taking into account the high number of endemic taxa reported from this underground ecosystem.

The centipede order Geophilomorpha is known to encompass about 1000–1250 extant species distributed on all continents except for Antarctica (Bonato et al. 2011, Bonato et al. 2014a). All species lack sight, have a more or less distinctly dorsoventrally flattened trunk, and are well adapted to an underground mode of life. Burrowing locomotion is typical for geophilomorphs but species that dwell on the surface are also known in the group. Some ‘extreme’ habitat adaptations are demonstrated, for example, by species that inhabit surge zones of seashores, periodically inundated forests in Amazonia or deserts.

Although geophilomorphs can occasionally be found in caves, very few of them seem to be highly adapted to this environment. Members of the genus *Thracophilus* Verhoeff, 1926 (Himantariidae) have been for example most often recorded from caves in Europe but none of the species exhibit particularly troglomorphic traits. *Ityphilus cavernicolus* (Matic, Negrea & Fundora Martínez, 1977) (Ballophilidae), found in several caves in Cuba, was speculatively presumed to be a troglobite or a regular troglophile (Matic et al. 1977, Lewis 1981), though no sound evidence for this has yet been presented. True troglobites were unknown for the order until 1999, when *Geophilus persephones* Foddai & Minelli, 1999 was described from the large cave system of Pierre Saint-Martin in southern France.

**Figure 1.**
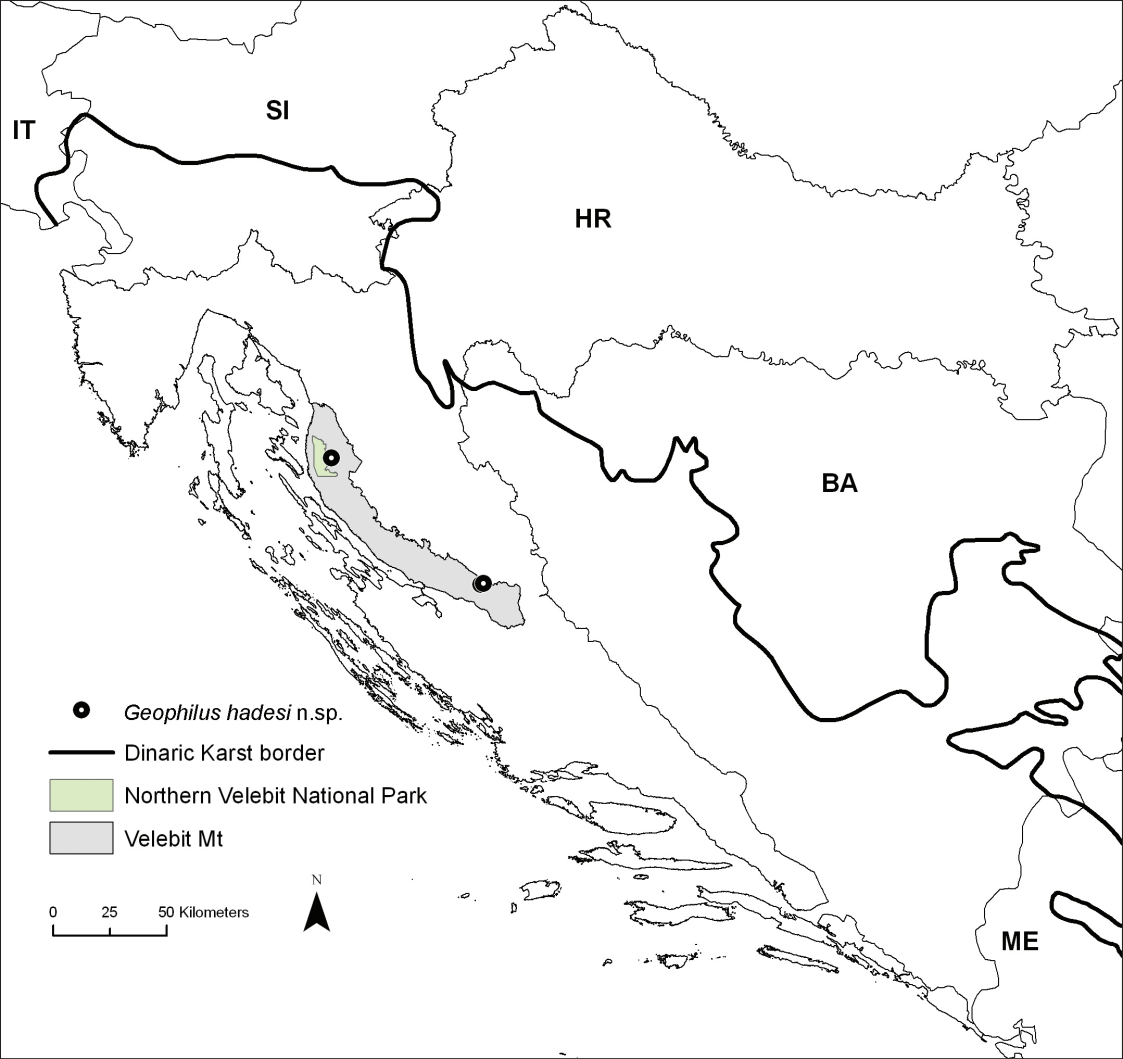
Map of Croatia showing the localities of *Geophilus hadesi* sp. n.

**Figure 2.**
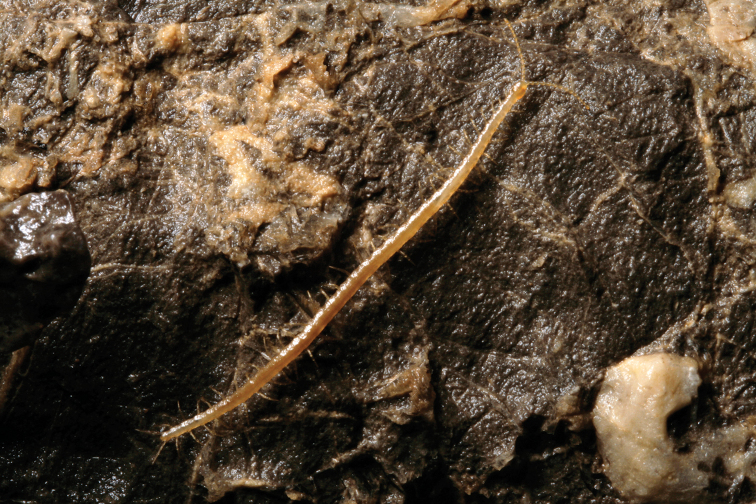
*Geophilus hadesi* sp. n. Habitus of male specimen (CBSS: CHP515). Photo taken *in situ* in the Lukina jama – Trojama cave system, at -980 m below the surface (Fig. 22), 1–3 August 2011. Photo credit: J. Bedek.

Here we describe a new troglobitic species of *Geophilus* Leach, 1814, *Geophilus hadesi* sp. n., characterized by relatively elongated trunk segments and appendages, including unusually long claws of the legs. The new species was recently found by Croatian biospeleologists at a great depth in some vertical caves in Velebit Mountain, central Croatia (Fig. 1). Specimens apparently belonging to the same species have been found also in the 15^th^ deepest cave in the world, Lukina jama – Trojama system (-1431 m) (Fig. 2), where one was collected in a large chamber at -980 m and another has been observed at an unreachable spot at -1100 m, the latter representing the world’s deepest record of a centipede known to date.

## Material and methods

Four specimens have been encountered during the exploration of three caves: Muda labudova, Munižaba and Lukina jama – Trojama cave system. With the exception of a specimen that was not reachable, the other three specimens were collected by hand, one from each cave, and preserved in 70% or 96% ethanol. Before collection, one of them was photographed *in situ* using a Canon 400D camera, with a 65 mm macro objective. All three specimens were compared by P.S. and A.K. using respectively a Zeiss and a Leica MZ16A stereomicroscopes. Additionally one specimen was documented by N.A. using scanning electron microscopy: all body parts were cleaned with ultrasound, transferred to 96% ethanol then to acetone, air-dried, mounted on aluminium stubs, coated with platinum/palladium and studied in a JEOL JSM-6335F scanning electron microscope. Images were edited in Adobe Lightroom 5 and Adobe Photoshop CS6. The specimens are shared between the Croatian Biospeleological Society Collection which is a part of Natural History Museum, Zagreb (CBSS) and the Naturrhistorisches Museum Wien (NHMW). All images included in this publication have been deposited in MorphBank (http://www.morphbank.net). Terminology for external anatomy follows Bonato et al. (2010).

## Results

### Taxonomy Family Geophilidae Leach, 1815 Genus *Geophilus* Leach, 1814

#### Geophilus hadesi

Taxon classificationAnimaliaGeophilomorphaGeophilidae

Stoev, Akkari, Komerički, Edgecombe & Bonato sp. n.

http://zoobank.org/1D7108A5-11D9-49C0-8D4E-EF39DBD80957

Figs 2 , 3–6 , 7–10 , 11–14 , 15–19

##### Material examined.

Holotype: female, 28 mm long, with 33 pairs of legs; Croatia, Zadarska županija (Zadar County), Southern Velebit Mountain, Crnopac massif, city Gračac, Munižaba cave, N44°15'57.4", E 15°52'09"; *circa* -250 m below the surface (cave entrance at 915 m a.s.l.); hand collected, 19 February 2011, leg. B. Jalžić (CBSS, collection code: CHP 532).

Paratype: female (damaged), approx. 22 mm long, with 33 pairs of legs; same area as the holotype, Muda labudova cave, N44°15'38.5", E15°51'18.2"; -500 m below the surface (cave entrance at 1020 m a.s.l.); hand collected, 2-3 July 2011, leg. J. Bedek (NHMW, collection code: NHMW8363, 3 SEM stubs)

Other specimen examined: male, approx. 27 mm long, with 33 pairs of legs; Croatia, Ličko-senjska županija (Lika-Senj County), Northern Velebit Mountains, Hajdučki i Rožanski kukovi Strict Reserve, Lukina jama – Trojama cave system, N44°46'01.6", E15°01'52.7"; -980 m below the surface (cave entrace at 1475 m a.s.l.); hand collected on boulder, 1–3 August 2011, leg. J. Bedek (CBSS: CHP515).

##### Origin of name.

The specific epithet derives from Hades, god of the underworld in Greek mythology and husband of Persephone, in analogy with the name of the only other known troglobite in the genus.

##### Diagnosis.

A species of *Geophilus* with a slender body, adult *circa* 2.2–2.8 cm in length; antennae *circa* 4.5–5 times as long as the head; second maxillary pretarsus very small, tubercle-like with a short tip; exposed part of the forcipular coxosternite more than 1.8 times as wide as long, coxopleural sutures strongly diverging forwards all along their length, chitin-lines incomplete; trunk metasternites elongate, with carpophagus pit; sternal pore-fields on both anterior and posterior parts of trunk; legs elongate, 33 pairs, with long claws; metasternite of the ultimate leg-bearing segment wider than long; coxal pores only on the ventral side, most of them close to the margin of the metasternite, also a single one isolated posteriorly; legs of the ultimate pair with claws.

From the other European species of *Geophilus* with a similarly low number of legs, it can be readily distinguished by a number of traits (see Table 1 and Discussion below).

**Table 1.** Comparison between *Geophilus hadesi* sp. n. and all other European congeners that have fewer than 37 pairs of legs. Data mainly from Bonato et al. (2014b). * The morphology of *Geophilus guanophilus* and *Geophilus minimus* is poorly known.

	*Geophilus hadesi* sp. n.	*Geophilus guanophilus* Verhoeff, 1939	*Geophilus minimus* Verhoeff, 1928	*Geophilus persephones* Foddai & Minelli, 1999	*Geophilus piae* Minelli, 1983	*Geophilus ribauti* Brölemann, 1908	*Geophilus richardi* Brölemann, 1904	*Geophilus truncorum* Bergsøe & Meinert, 1866
Distribution	Velebit	Salento peninsula	SW Alps, Sardinia	Pyrenees	Sardinia, Sicily	Pyrenees, Massif Central, SW Alps	W Alps, Sardinia, Italian peninsula, Sicily, Ionian islands	central and northern Europe
Recorded in caves	yes	yes	no	yes	no	no	no	no
Body length (maximum recorded)	28 mm	18 mm	9.5 mm	16.2 mm	11 mm	<20 mm	10 mm	20 mm
Antennae length / head width	4–5	?	<3	6–7	<3	<3	<3	<3
Second maxillae: pretarsus: shape	stout tubercle with small tip	gradually tapering, curved	stout tubercle with small tip?	stout tubercle with small tip	stout tubercle with small tip	gradually tapering, curved	gradually tapering, curved	gradually tapering, curved
Forcipular coxosternite: width / length of exposed part	>1.8	?	<1.8	<1.8	<1.8	<1.8	<1.8	<1.8
Legs: number of pairs (recorded values)	33	35, 37	33, 35, 37	29	35, 37	33, 35, 37	29, 31, 33	37, 39, 41
Carpophagus pit	yes	yes	?	yes	yes	yes	no	yes
Pore-fields	present (also in posterior part)	absent	present (only in anterior part)	present (also in posterior part)	present (also in posterior part)	absent	absent	absent
Coxal pores: number on each side (maximum recorded)	6	3	4	4	2	4	2	2
Coxal pores: isolated posterior pore	yes	?	?	no	no	no	no	no

##### Description of the holotype.

Length 28 mm (damaged paratype: *circa* 22 mm).

**Figures 3–6.**
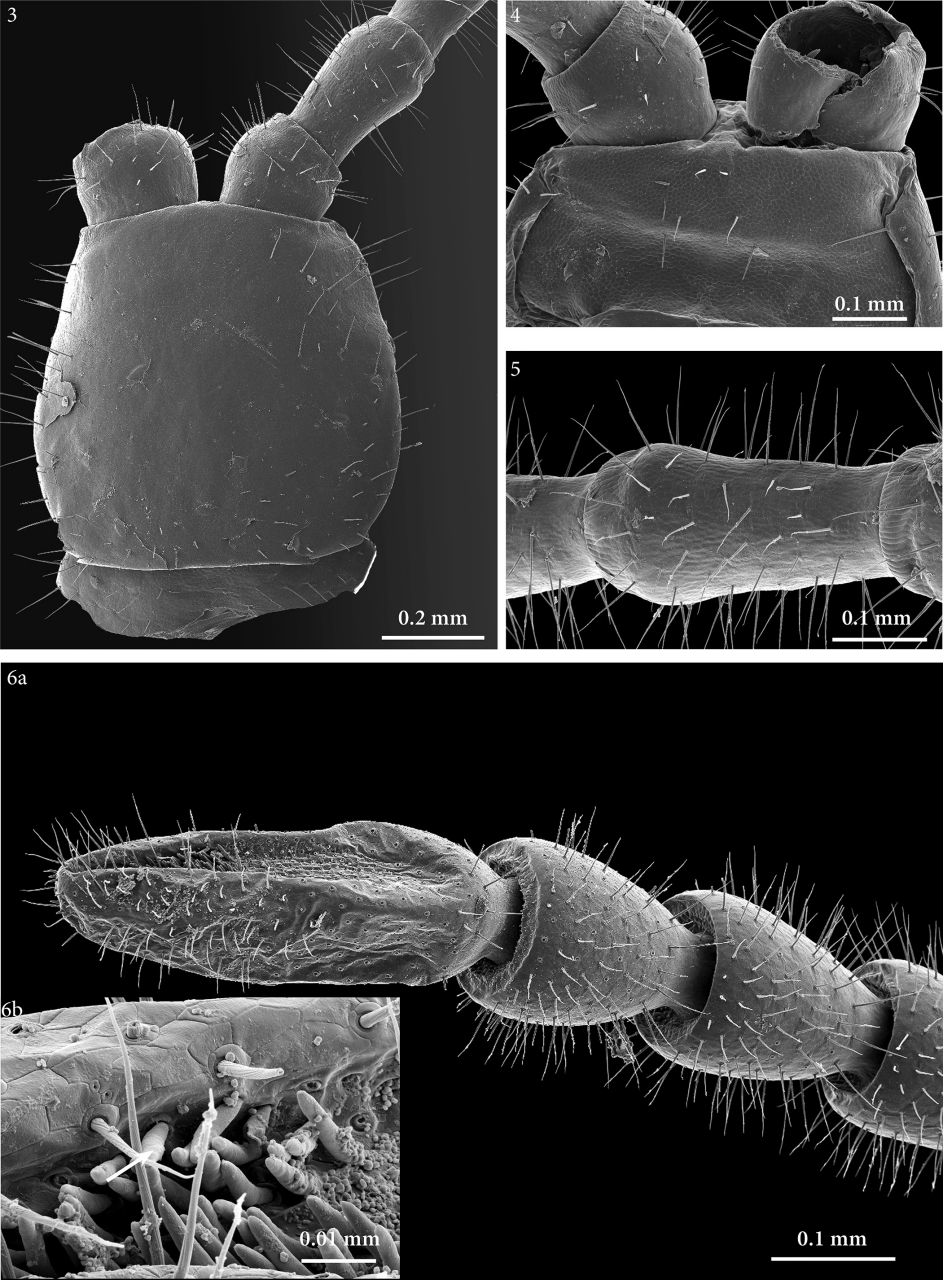
*Geophilus hadesi* sp. n. **3** cephalic capsule, dorsal **4** clypeus, ventral **5** right antenna, article IV, dorsal **6a** right antenna, ultimate articles, dorsal **6b** right antenna, close up of sensilla basiconica cluster, mesal. SEM micrographs taken from female paratype NHMW8363 from cave Muda labudova.

*Cephalic plate.* Cephalic plate as long as wide (also in paratype: Fig. 3), anterior margin slightly angulated, lateral margins evidently convex and convergent forward for their anterior two thirds, posterior margin almost straight; transverse suture absent. Most setae 100–120 µm long.

*Antennae*. *Circa* 4.6 times as long as the maximum width of the head (also in paratype: Figs 5, 6); all but first article longer than broad, proportions between articles: V=VI>VII=IV>VIII>IX>III>II=X>XI>I>XIV>XIII>XII. Ultimate article twice as long as penultimate (also in paratype: Fig. 6a). Antennae densely covered with setae, which are nearly as long as antennal breadth on the basal articles, gradually shorter and more dense from basal towards the distal articles. Apical sensilla slender, spear-like, *circa* 14 μm long, slightly narrowing towards the tip. Club-like sensilla (sensilla basiconica) in two groups, one on the internal side and the other on the external side on article XIV (also in paratype: Fig. 6b).

*Clypeus*. Uniformly areolate, without finely areolate clypeal areas; three pairs of setae including a paramedian pair close to the anterior margin and two pairs of larger setae in the middle of clypeus (also in paratype: Fig. 4).

**Figures 7–10.**
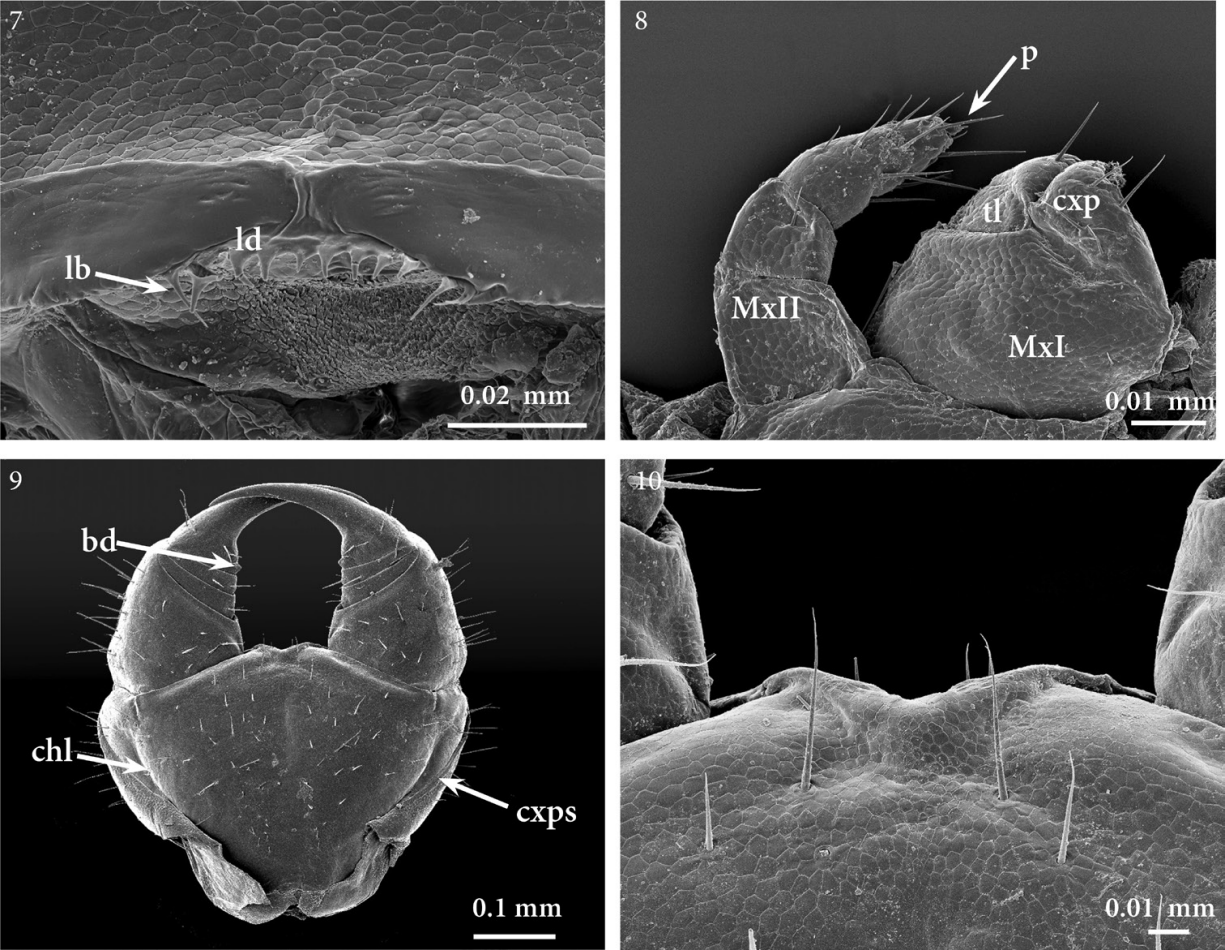
*Geophilus hadesi* sp. n. **7** intermediate part of labrum, ventral **8** right half of the maxillary complex, ventral **9** forcipular segment, ventral **10** close up of forcipular coxosternite, ventral. SEM micrographs taken from female paratype NHMW8363 from cave Muda labudova. Abbreviations: labral bristle (*lb*), labral denticle (*ld*), first maxillae (*MxI*), second maxillae (*MxII*), first maxillary telopodite (*tl*), first maxillary coxal projection (*cxp*), second maxillary pretarsus (*p*), coxopleural suture (*cxps*), chitin-line (*chl*), basal denticle of tarsungulum (*bd*).

*Labrum*. About eight triangular denticles on the intermediate part of the margin, and some longer and thinner bristles on the lateral parts (also in paratype: Fig. 7).

*Maxillary complex*. First maxillae: coxosternite entire; coxal projections subtriangular, bearing four large setae and one subapical spine-like seta; telopodites almost the size of the coxal projections, with the intermediate articulation weakly distinct; lappets apparently absent or very short (also in paratype: Fig. 8). Second maxillae: coxosternite entire, uniformly areolate, the anterior margin widely concave, with setae close to the anterior margin; telopodite composed of three articles, gradually narrowing towards the tip; basal article with three long setae close to the inner margin, article 2 with one seta, article 3 with 13–15 setae; pretarsus small, tubercle-like with a small apical tip (also in paratype: Fig. 8).

*Forcipular segment*. Forcipules, when closed, not exceeding the anterior margin of the head. Tergite wider than long, lateral margins evidently convex. Ventrally exposed part of the coxosternite 1.8–1.9 times as wide as long, anterior margin without denticles (also in paratype: Figs 9, 10); coxosternal denticles replaced by a prominent coxosternal ridge, with a pair of long setae exceeding the coxosternal margin (Fig. 10); coxopleural sutures strongly diverging forwards all along their length; chitin-lines incomplete and pointing lateral from the condyles. Trochanteroprefemur with external side almost twice as long as the internal side. Tarsungulum with a small denticle at the base, abruptly narrowing and bent basad, then gradually tapering. No denticles on the other forcipular articles.

**Figures 11–14.**
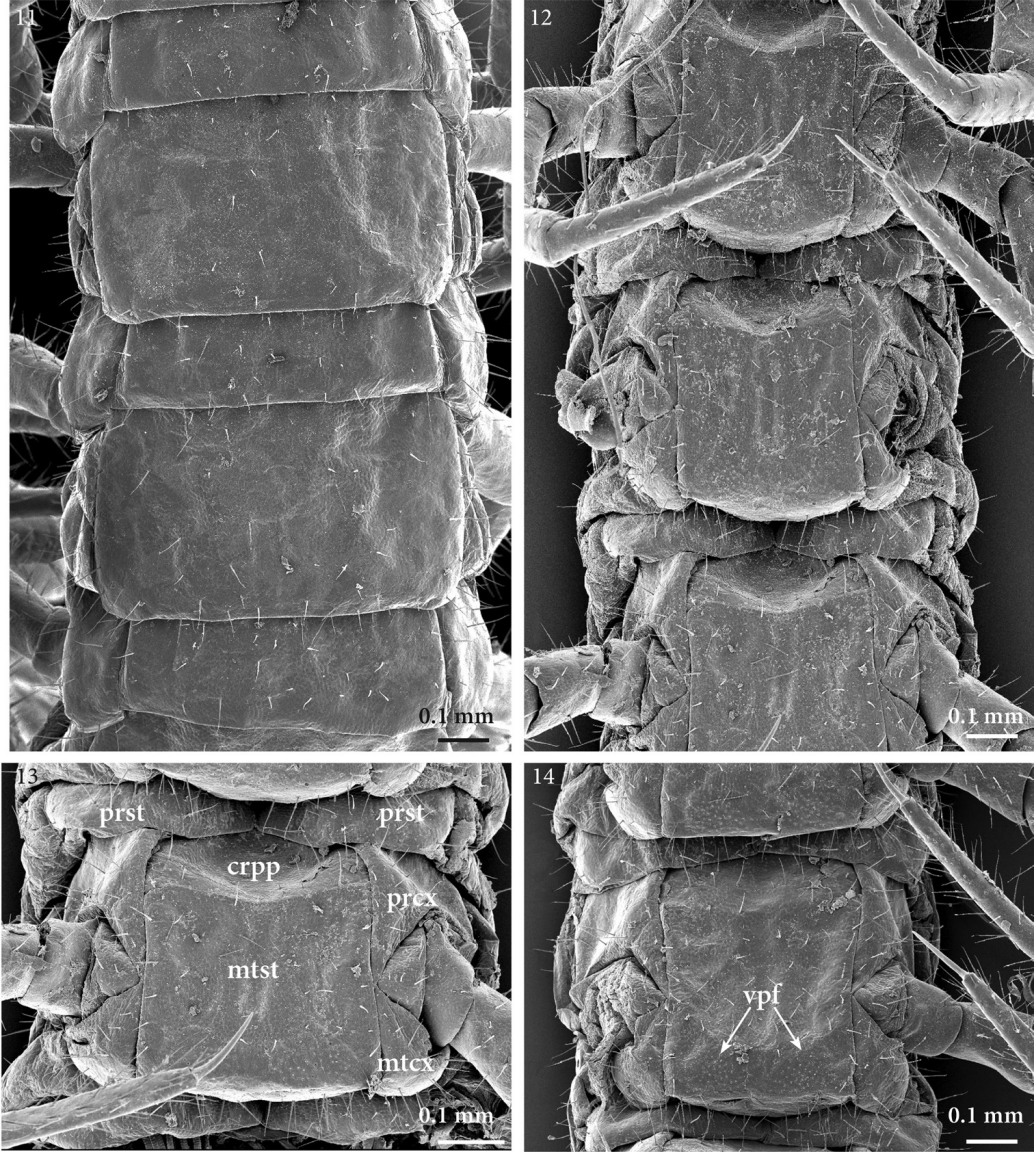
*Geophilus hadesi* sp. n. **11** midbody tergites, dorsal **12** sternites of leg-bearing segments 6–8, ventral **13** leg-bearing segment 8, ventral **14** leg-bearing segment 25, ventral. SEM micrographs taken from female paratype NHMW8363 from cave Muda labudova. Abbreviations: presternites (*prst*); carpophagus pit (*crpp*), procoxa (*prcx*), metacoxa (*mtcx*), metasternite (*mtst*), ventral pore-field (*vpf*).

**Figures 15–19.**
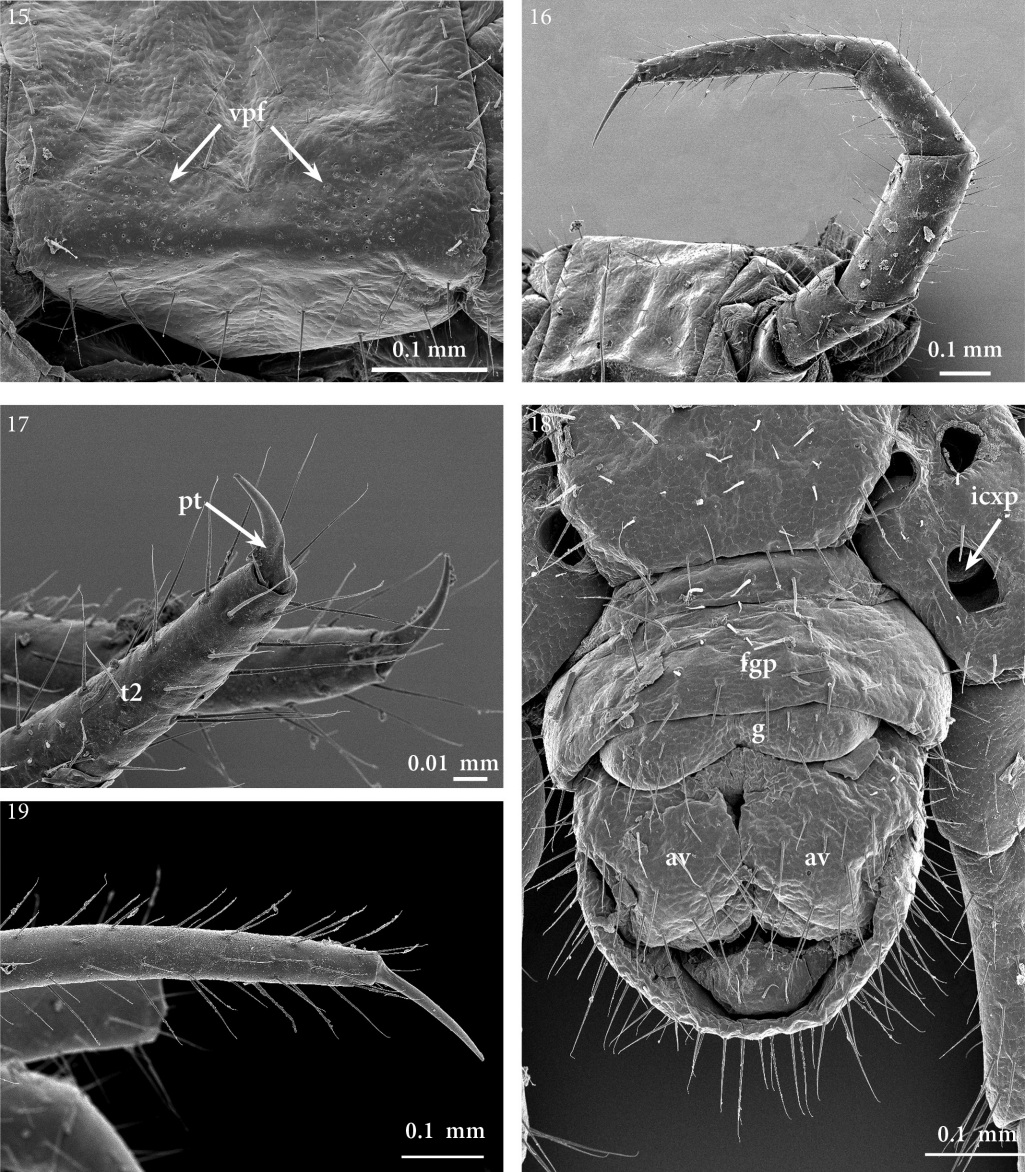
*Geophilus hadesi* sp. n. **15** close up of metasternite of leg-bearing segment 26, ventral **16** left leg 8, anterio-lateral **17** close up of the tip of the right ultimate leg, external view **18** postpedal segments, ventral **19** close up of pretarsus and tarsus of midbody leg, lateral. SEM micrographs taken from female paratype NHMW8363 from cave Muda labudova. Abbreviations: tarsus 2 (*t2*), pretarsus (*pt*), isolated coxal pore (*icxp*), anal valve (*av*), first genital pleurosternite (*fgp*), gonopods (*g*).

*Leg-bearing segments*. A total of 33 leg-bearing segments (also in paratype). Tergites wider than long (Fig. 11), tergite 1 slightly wider than metatergite 2. Metasternites longer than wide, both in the anterior and the posterior part of trunk. Those in the anterior part of the trunk, with the exception of the first segment, with anterior carpophagus pits taking up one third of the length of the metasternite (also in paratype: Figs 12, 13); pits decreasing in size towards the body end to almost vanishing in the last five segments (Fig. 14). Pore-fields present on most part of the metasternites (also in paratype: Figs 12–15): a single transverse band along the posterior margin of each metasternite, appearing as two paramedian groups on the metasternites of the posterior half of the trunk, and poorly visible posterior to leg-bearing segment 26. Legs distinctly longer than the breadth of the body; their length increases towards leg pairs 25–26, then gradually decreasing until pair 32; leg 1 shortest. Leg claws very slender (Figs 16, 17, 19), more than 6 times as long as broad at the basis, with two long and pointed accessory spines.

**Figure 20.**
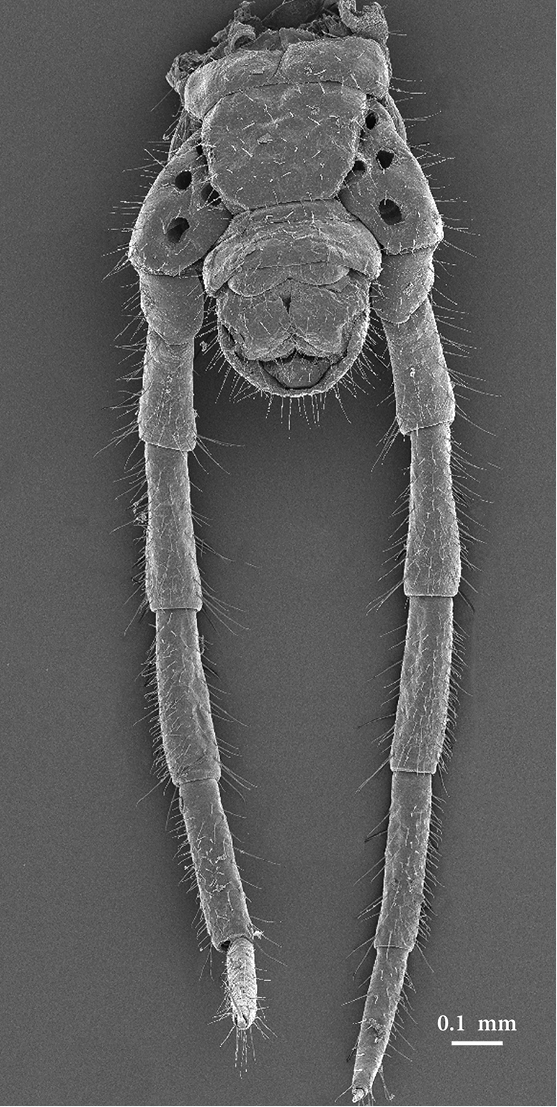
Ultimate leg-bearing segment and postpedal segments, ventral. Photo taken from female paratype NHMW8363 from cave Muda labudova.

*Ultimate leg-bearing segment*. Metasternite sub-trapezoidal, *circa* 1.3 times as wide as long, lateral margins converging posteriorly; posterior margin narrower than anterior; setae uniformly scattered. Coxopleura moderately swollen, reaching backward approximately two thirds of first genital pleurosternite. Coxal organs opening through distinct pores, all on the ventral side, mostly close to the lateral margins of the metasternite, 5+6 (5+5 in paratype). Coxal pores generally large, one lying in the middle of the ventral side of the posterior part of the coxopleuron, somewhat apart from the rest (also in paratype: Figs 18, 20). Telopodites of the ultimate pair almost as long as legs 25-26. Proportions between the lengths of the leg articles: trochanter<prefemur<coxa=tarsus2<femur=tibia=tarsus1; claws shorter than those of preceding legs, accessory spines distinctly shorter.

*Postpedal segments*. First genital pleurosternite entire. Gonopodal lamina distinctly bilobed. Anal pores present (also in paratype: Fig. 18).

## Discussion

### On the taxonomic position of *Geophilus hadesi* sp. n.

Although relationships between the species in the genus *Geophilus* are still largely unclear, *Geophilus hadesi* sp. n. is probably related to a subgroup of species sharing a putatively modified shape of the second maxillary pretarsus, which resembles a stout tubercle with a short spine rather than an elongate curved claw. Three genus group names are available for this group, i.e. *Orinomus* Attems, 1895, *Orinophilus* Cook, 1896, and *Cyphonychius* Verhoeff, 1928, but their nomenclatural status is yet to be evaluated. Most of these species inhabit western and southern Europe, but *Geophilus alpinus* Meinert, 1870 and *Geophilus oligopus* (Attems, 1895) have been recorded also from the Balkan peninsula (Bonato et al. 2014b). Among all these species, *Geophilus oligopus*, *Geophilus persephones* and *Geophilus piae* especially resemble *Geophilus hadesi* sp. n. in the reduced number of segments, with their leg pairs varying mostly from 29 (recorded in *Geophilus persephones*) to 39 (recorded in *Geophilus oligopus*), but they are smaller than *Geophilus hadesi* sp. n., with a length not reaching 2 cm. The other known troglobitic species *Geophilus persephones* shares with the new species a number of distinctive traits (including elongate antennae, walking and ultimate legs), but this may be due to convergence to a troglomorphic habitus. Another shared trait is the shape of the labrum devoid of separation between mid and lateral parts.

### On the habitat of *Geophilus hadesi* sp. n.

Besides its troglomorphic appearance, the fact that all the four specimens (three collected) were recorded in the same type of habitat suggests that *Geophilus hadesi* sp. n. is a highly adapted cave animal.

**Figure 21.**
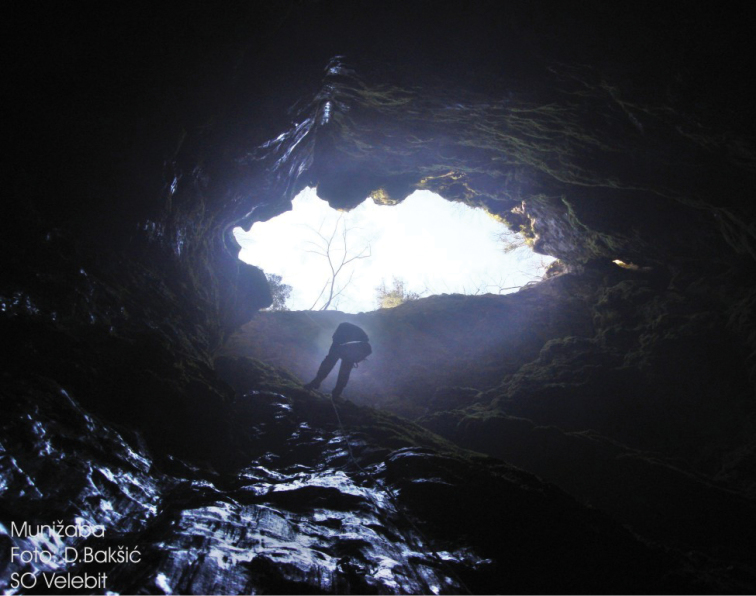
The entrance of cave Munižaba. Photo: D. Bakšić.

The holotype and paratype were found in two caves, Munižaba and Muda labudova, both situated in Crnopac massif, Southeastern Velebit Mountain. Munižaba cave is the most voluminous cave in Croatia, with the entrance dimensions of 30 × 35 m and a vertical drop of 200 meters (Fig. 21). The cave has been explored to the depth of -510 m and up to 9715 m in horizontal length. A specimen of *Geophilus hadesi* was found under the entrance shaft at a depth of *circa* -250 m, but further details are not known. Muda labudova is a 680 m deep cave with low temperatures and a lot of snow in the first 200 m of depth. There a specimen was found at *circa* -500 m while moving on the cave wall. Detailed description of the morphology, genesis and climatic conditions of the Munižaba and Muda labudova caves, along with their maps and associated fauna, can be found in Casale et al. (2012).

**Figure 22.**
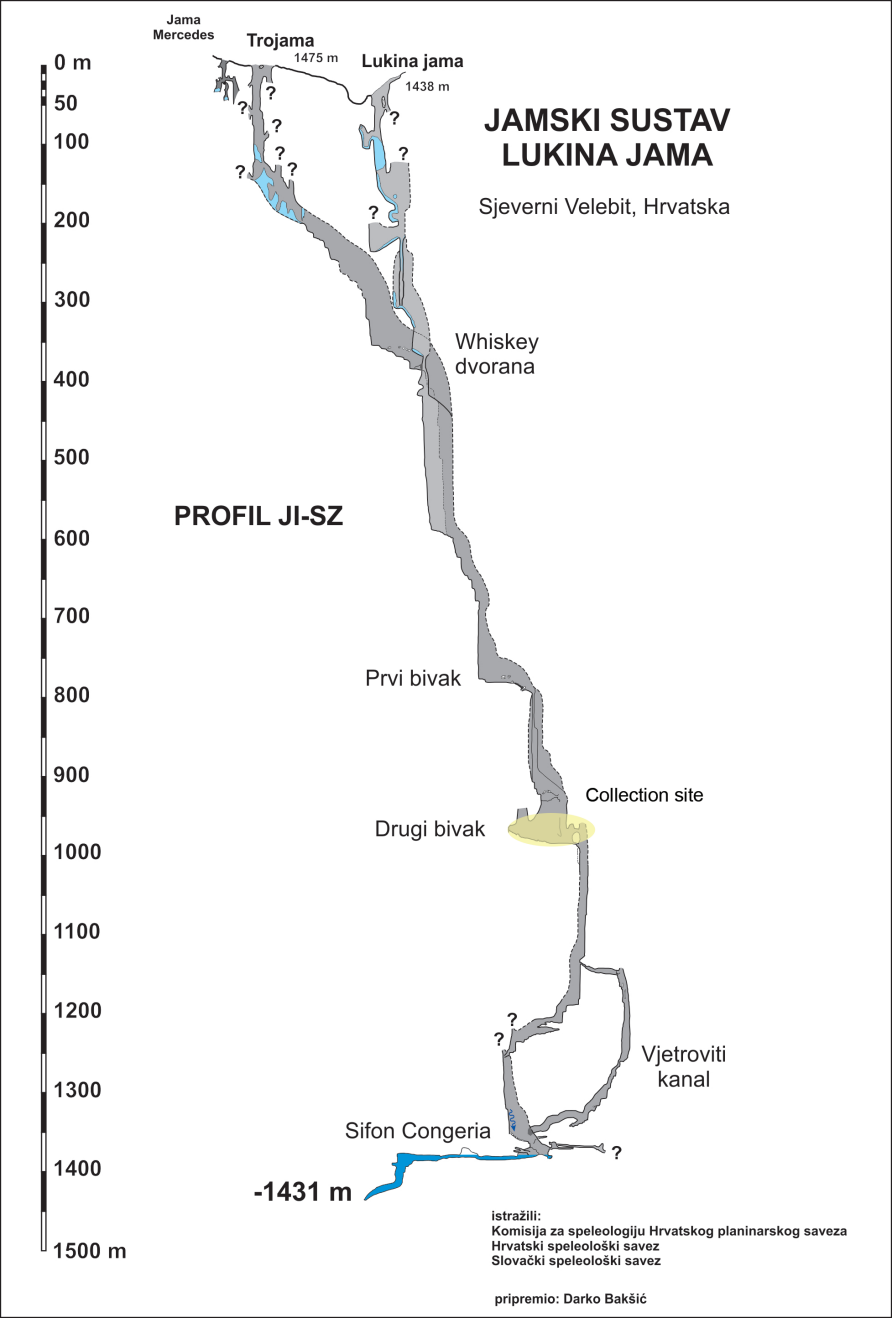
A map of Lukina Jama – Trojama cave system. Collection site marked in yellow.

**Figure 23.**
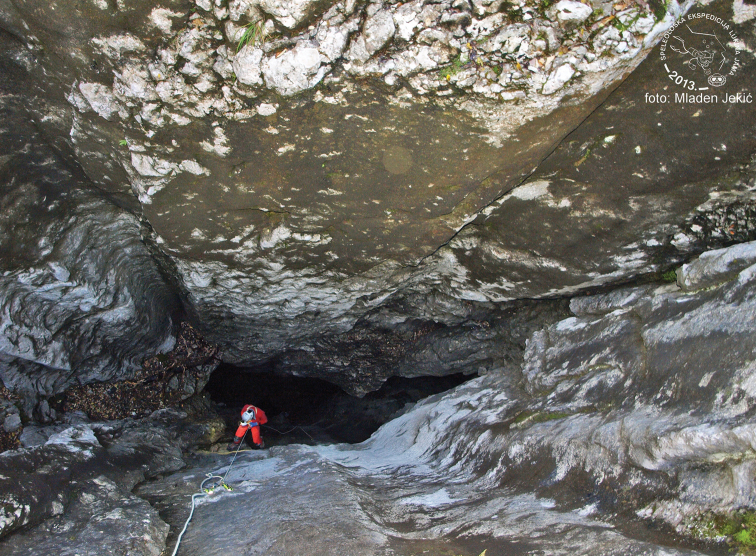
Entrance of cave Lukina jama. Photo: M. Jekić

Another two specimens apparently belonging to the same species were found in the Lukina jama – Trojama cave system, which is 1431 meters deep and is currently the deepest cave in the Dinaric Karst and the 15th deepest cave in the world (see http://www.caverbob.com/wdeep.htm and Bakšić et. al. 2007) (Fig. 22). The cave is situated inside the Hajdučki i Rožanski kukovi Strict Reserve, Velebit National Park, and has two entrances, which connect at 558 m of depth, the upper one (Trojama) being at 1475 m above sea level (Fig. 23). Both entrances are blocked with snow and ice blocks during most of the year. In the cave system two temperature gradients can be determined; one from the entrance to a depth of -200 m, and a second from -200 m to the bottom of the cave. The entrance part down to -200 m is under influence of outside conditions and the ice and the temperature gradually lowers to -5.8 ˚C, while from -200 m to the bottom it slowly rises, reaching 3.3–4.4 ˚C in the chamber at -980 m and 5.0 ˚C at the bottom (Paar et al. 2013). There is a constant water flow from a depth of -550 meters to the bottom and measurements have confirmed that the water level in the cave rises over 100 meters above the bottom during high water stands (Bedek et al. 2012). A specimen was found in Lukina jama – Trojama cave system in 2011 in a vast chamber at -980 m while moving slowly across a large boulder close to a water flow (Fig. 2). A second specimen was observed at *circa* -1100 m while moving on the wall and was out of reach of the collector. The latter represents the world’s deepest record of Chilopoda as a whole.

A total of 54 animal taxa have been recorded in Lukina jama – Trojama cave system until 2013, including four new genera and seven new species (Čuković et al. 2014, present study). Terrestrial taxa are dominant (37 species), and include 19 troglobites, while out of 17 aquatic taxa 13 are stygobites. Certain taxa, i.e. *Velebitodromus smidai* (Casale, Giachino & Jalžić, 2004) (Coleoptera), *Congeria jalzici* Bilandžija & Morton, 2013 (Bivalvia) and *Zospeum tholossum* Weigand, 2013 (Gastropoda) exhibit high level of adaptation to the cave environment. A tentative list of all associated fauna is presented in Table 2.

**Table 2.** List of animal taxa found in Lukina jama – Trojama cave system (Čuković et al. 2014, present study).

Taxonomic group	Taxa
Porifera	*Eunapius subterraneus* Sket & Velikonja, 1984
Rotifera	*Keratella quadrata* (Müller, 1786)
Gastropoda	*Lanzaia* sp., *Hauffenia* sp., *Zospeum subobesum* Bole, 1974, *Zospeum tholossum* Weigand, 2013
Bivalvia	*Congeria jalzici* Bilandžija & Morton, 2013
Polychaeta	*Marifugia cavatica* Absolon & Hrabe, 1930
Hirudinea	*Croatobranchus mestrovi* Kerovec, Kučinić & Jalžić, 1999
Acari	*Rhagidia* sp., *Nicoletiella* sp.
Pseudoscorpiones	*Neobisium svetovidi* Ćurćić, 1988
Isopoda	*Proasellus* sp., *Alpioniscus* sp. n.
Amphipoda	*Niphargus arbiter* G. Karaman, 1985, *Niphargus brevirostris* Sket, 1971, *Niphargus croaticus* (Jurinac, 1888), *Niphargobates* sp.
Decapoda	Troglocaris cf. kapelana, *Troglocaris* sp.
Diplopoda	*Brachydesmus* sp., *Haasia stenopodium* (Strasser, 1966)
Chilopoda	*Geophilus hadesi* sp. n.
Collembola	Isotomidae gen. n., sp. n., *Disparrhopalites* sp. n., Tullbergiidae gen. sp.
Diplura	Plusiocampa (Stygiocampa) sp. n.
Coleoptera	*Astagobius angustatus* Schmidt, 1852, *Spelaeodromus pluto* (Reitter, 1881), *Velebitodromus smidai* (Casale, Giachino & Jalžić, 2004), *Laemostenus* sp.
Diptera	Chironomidae gen. n., sp. n., Mycetophilidae gen. sp.
Chiroptera	*Myotis mystacinus* (Kühl, 1817)

The three caves have been biospeleologically investigated on numerous occasions during the past 10 years, but only three centipede specimens have been collected so far. One of the reasons is the difficulty of collecting in vast cave systems with large chambers and passages which provide an enormous habitat surface that is impossible to examine thouroughly. Collecting effort is also to be taken into consideration. In 2010 and 2013 caving expeditions in the Lukina jama – Trojama cave system included teams of biologists spending a minimum of two days in the chamber at -980 m, with more than 36 man-hours in 2010 (Bedek et al. 2012) and over 128 man-hours in 2013. The result was only one observed specimen at -1100 m. However in 2011, when a specimen was collected, only one biologist was in the chamber collecting for 18 hours. Unlike in 2011, in 2010 and 2013 baited pitfall traps were placed throughout the cave system and the area around them was examined in case a predator such as a centipede or beetle was attracted to the possibility of nearby prey, but no centipede was found. Such were also the cases with some Araneae and Opiliones species recorded in the cave system and can only be explained by “collectors’ luck”.

### On the subterranean biodiversity of Velebit Mountain

Velebit stretches over 145 km and is situated in the Croatian Dinaric Karst area (Paar et al. 2013), which is considered a remarkable hot spot of subterranean diversity (Casale et al. 2012). The mountain is divided into four regions: Northern, Middle, Southern and Southeastern (Bognar 1994). There is a significant difference in karst morphology between the regions. Northern Velebit hosts a large number of extremely vertical and deep caves (Paar et al. 2013), including all the three caves in Croatia deeper than 1000 m. Towards the south, caves are more horizontal. Southeastern Velebit is an area of Crnopac massif, where caves reach depths of over 700 m but create complex systems. The longest cave in the Dinaric Arc, the Kita Gačešina – Draženova puhaljka cave system, over 27 km in length, is surrounded by more caves and systems awaiting investigation. Currently there is a perspective of connecting this system with Munižaba into a unique system over 37 km long (Bakšić and Rakovac 2014). Despite the differences in karst morphology, the conditions in the caves in Velebit are similar, with stable low temperatures and an average of 90–100% humidity.

The lack of finds in Middle and South Velebit is possibly due to undersampling in these areas, which have been much less investigated speleologically and biologically than the caves in Northern Velebit and the Crnopac area.

Some subterranean species are endemic to caves on Velebit Mountain (e.g., *Croatobranchus mestrovi* Kerovec, Kučinić & Jalžić, 1999) while for others (e.g., Neobisium (Pennobisium) stribogi Ćurčić, 1988) it is only a part of their distribution range. Certain areas of Croatian Dinaric Karst are still poorly investigated, e.g. Biokovo Mountain in the south of Croatia, where caves have so far been explored to -831 m of depth. Future research in these undersampled areas will provide a better insight into the distribution area and possible habitat preferences of the species.

While several highly specialized cave invertebrates are known to inhabit both caves, Muda labudova and Munižaba, thus showing similar distribution patterns (Casale et al. 2012, Antić et al. 2014), still little is known about the faunal links between caves in the northern part of Velebit, such as the Lukina jama – Trojama cave system, and those in the south. *Geophilus hadesi* sp. n. offers an example for investigating the history of underground colonization in these two areas.

## Supplementary Material

XML Treatment for Geophilus hadesi
